# Ensemble machine learning-based recommendation system for effective prediction of suitable agricultural crop cultivation

**DOI:** 10.3389/fpls.2023.1234555

**Published:** 2023-08-10

**Authors:** Mahmudul Hasan, Md Abu Marjan, Md Palash Uddin, Masud Ibn Afjal, Seifedine Kardy, Shaoqi Ma, Yunyoung Nam

**Affiliations:** ^1^ Department of Computer Science and Engineering, Hajee Mohammad Danesh Science and Technology University, Dinajpur, Bangladesh; ^2^ School of Information Technology, Deakin University, Geelong, VIC, Australia; ^3^ Department of Applied Data Science, Noroff University College, Kristiansand, Norway; ^4^ Artificial Intelligence Research Center (AIRC), Ajman University, Ajman, United Arab Emirates; ^5^ Department of Electrical and Computer Engineering, Lebanese American University, Byblos, Lebanon; ^6^ Department of ICT Convergence, Soonchunhyang University, Asan, Republic of Korea

**Keywords:** crop production, crop prediction, agricultural data processing, machine learning, ensemble learning

## Abstract

Agriculture is the most critical sector for food supply on the earth, and it is also responsible for supplying raw materials for other industrial productions. Currently, the growth in agricultural production is not sufficient to keep up with the growing population, which may result in a food shortfall for the world’s inhabitants. As a result, increasing food production is crucial for developing nations with limited land and resources. It is essential to select a suitable crop for a specific region to increase its production rate. Effective crop production forecasting in that area based on historical data, including environmental and cultivation areas, and crop production amount, is required. However, the data for such forecasting are not publicly available. As such, in this paper, we take a case study of a developing country, Bangladesh, whose economy relies on agriculture. We first gather and preprocess the data from the relevant research institutions of Bangladesh and then propose an ensemble machine learning approach, called K-nearest Neighbor Random Forest Ridge Regression (KRR), to effectively predict the production of the major crops (three different kinds of rice, potato, and wheat). KRR is designed after investigating five existing traditional machine learning (Support Vector Regression, Naïve Bayes, and Ridge Regression) and ensemble learning (Random Forest and CatBoost) algorithms. We consider four classical evaluation metrics, i.e., mean absolute error, mean square error (MSE), root MSE, and *R* ^2^, to evaluate the performance of the proposed KRR over the other machine learning models. It shows 0.009 MSE, 99% *R* ^2^ for Aus; 0.92 MSE, 90% *R* ^2^ for Aman; 0.246 MSE, 99% *R* ^2^ for Boro; 0.062 MSE, 99% *R* ^2^ for wheat; and 0.016 MSE, 99% *R* ^2^ for potato production prediction. The Diebold–Mariano test is conducted to check the robustness of the proposed ensemble model, KRR. In most cases, it shows 1% and 5% significance compared to the benchmark ML models. Lastly, we design a recommender system that suggests suitable crops for a specific land area for cultivation in the next season. We believe that the proposed paradigm will help the farmers and personnel in the agricultural sector leverage proper crop cultivation and production.

## Introduction

A constructive agricultural environment and fertile land make agriculture the leading economic sector for a developing country whose economy relies on agriculture. Agriculture is associated with producing essential food crops and industrial raw materials. One of the most critical aspects of the development cycle of a country is the capacity to produce food using the unfavorable environment and limited agricultural land (Goldstein et al., 2017). Experts believe that land fertility has reduced to a certain extent over time, affecting the crop production amount (Van Klompenburg et al., 2020). In this paper, we consider the case study of a developing country, Bangladesh, whose economy relies on agriculture. According to the Bangladesh Rural Advancement Committee (BRAC), the agricultural land in Bangladesh is shrinking by 1% annually, while the population is growing by 1.2% annually (Das et al., 2022). In addition, the farmers do not get the actual price due to the lack of knowledge of the estimated crop production. This concern demotivates the farmers, which has a long-term negative impact on the agriculture sector. To alleviate this issue, proper planning of the best crop production in terms of correctly predicting crop production for the upcoming year can be provided to the farmers. The ability to accurately predict crop yields has become essential for farmers to make rational choices (Jansson et al., 2021). Various aspects, such as soil type, weather, and crop management practices, are taken into account to estimate the number of crops that may be grown in a particular area. Effective prediction helps to generate an estimation of crops that helps the government to take long-term and short-term policies to minimize food shortages and import–export plans based on the agriculture sector (Zhang et al., 2019). It also significantly impacts the economy of an agricultural-based country like our study area. Machine learning (ML) offers the most effective tool to predict the dependent variables (i.e., crop production) using the independent variables (i.e., the factors that regulate crop production) (Jayalakshmi and Gomathi, 2020; Ahmed et al., 2022; Li et al., 2022; Monteiro et al., 2022). In this paper, we investigate the simple but effective ML approaches to propose an ensemble ML approach toward accurately predicting the agricultural crop production of Bangladesh.

Bangladesh is a country with six seasons, which enables producing different kinds of crops over the year (Uddin et al., 2019) while its main crops are rice, wheat, and potato. Rice is the staple crop, and it can be cultivated in three different seasons where the rice varieties are Aus, Aman, and Boro. Potato and wheat are the second and third most important crops, respectively. As such, we predict the production of these five major crops (Aus rice, Aman rice, Boro rice, potato, and wheat) for the upcoming season based on the environmental data (i.e., rainfall, humidity, minimum and maximum temperature, sunshine, wind speed, and cloud coverage of a specific zone), cultivation area, and previous production data. We use historical data from 1969 to 2021 of different districts of Bangladesh (Campbell et al., 2020) and collect these raw data from different respective government organizations. In particular, we gather the raw data from the yearbooks of the Bangladesh Meteorological Department (BMD), Bangladesh Agricultural Development Corporation (BADC), Bangladesh Rice Research Institute (BRRI), and Bangladesh Bureau of Statistics (BBS). After that, we investigate the classical ML algorithms, i.e., Support Vector Regression (SVR), Naïve Bayes (NB), and Ridge Regression (RR), and ensemble ML algorithms, i.e., Random Forest (RF) and CatBoost (CB). Then, we propose an ensemble ML paradigm combining K-Nearest Neighbors (KNN), RF, and RR, termed K-nearest neighbors Random Forest Ridge regression (KRR), to effectively predict the production of the crops. Finally, we construct a recommender system that suggests suitable crops for a given land area for cultivation in the next season. The main contributions of this paper are summarized below.

- Development (collection, reformation, and data processing) of an ML trainable crop dataset containing environmental, cultivation area, and previous production data for predicting five major crops (Aus rice, Aman rice, Boro rice, potato, and wheat);
- Investigation and rigorous study of setting up a baseline ML system with effective ML and ensemble ML models for predicting crop production more efficiently;
- Design of a novel ensemble ML algorithm to accurately predict the production of the crops and Diebold–Mariano (DM) testing of the designed ensemble ML model to illustrate its significance and superiority over the benchmark ML and ensemble ML algorithms; and
- Designing a recommendation system for suggesting suitable crops for cultivating in a specific region in the next season among the contemporary crops.

The rest of this paper is structured as follows. In the *Related work* section, we discuss and compare the related works on crop production prediction. The *Proposed paradigm* section describes the overall idea and development of the proposed crop production prediction and recommendation paradigm. In the *Methods and measurements* section, we discuss the methods and materials for dataset generation, existing ML and ensemble ML methods, and our proposed ensemble ML approach. The experiments and results are explained and analyzed in the *Experiment and result analysis* section. The *Crop recommender system* section demonstrates the recommender system design for suggesting suitable crop cultivation in the upcoming season, while the *Conclusion* section summarizes and concludes the observations and findings. All the abbreviations used in this paper are listed in **Table 1**.

**Table 1:** List of the abbreviations used in the paper.

Abbreviations	Full Form	Abbreviations	Full Form
KRR	K-nearest Neighbor Random Forest Ridge Regression	BRAC	Bangladesh Rural Advancement Committee
ML	Machine Learning	BMD	Bangladesh Meteorological Department
BADC	Bangladesh Agricultural Development Corporation	BRRI	Bangladesh Rice Research Institute
BBS	Bangladesh Bureau of Statistics	SVR	Support Vector Regression
NB	Naive Bayes	RR	Ridge Regression
RF	Random Forest	CB	CatBoost
KNN	K-Nearest Neighbors	DM	Diebold–Mariano
ARIMA	Auto-regressive Integrated Moving Average	SVM	Support Vector Machine
APC	Average Pearson Correlation	CV	Coefficient of Variance
NN	Neural Network	LR	Logistic Regression
MSE	Mean Square Error	RMSE	Root Mean Square Error
MAE	Mean Absolute Error	RR	Ridge Regression
DT	Decision Tree	DL	Deep Learning

## Related work

Various applications of ML models in agriculture have been listed, such as crop yield prediction, weather forecasting, smart irrigation system, crop disease prediction, and deciding minimum support price (Al-Gaadi et al., 2016; Nandy and Singh, 2020; Sharma et al., 2020; Cravero and Sepulveda, 2021). Moreover, in order to achieve accurate predictions, researchers used the supervised ML algorithms for crop production prediction in (Kaur, 2016; Shehadeh et al., 2021). The decision tree (DT) classifier has been used to create predictions of yield and cropland temperature in (Lee and Moon, 2014) (Bagis et al., 2012). KNN and ID3 (a variant of DT) were applied to analyze the crop production of the previous year (Charbuty and Abdulazeez, 2021). Many researchers are using statistical models like Auto-regressive Integrated Moving Average (ARIMA) and ML model Support Vector Machine (SVM) for predicting crop production (Sujjaviriyasup and Pitiruek, 2013). On the other hand, time series analysis has been applied in order to predict the production and the price of crops and vegetables. The aim was to identify a time series function, which might identify patterns and seasonality in specific vegetables, as well as explore supply and demand variables (Bagis et al., 2012) (Jha and Sinha, 2013) (Young, 2019). In addition, many researchers proposed a methodology that uses Average Pearson Correlation (APC) and Coefficient of Variance (CV) to determine indications that reveal crop price fluctuation (Pereira et al., 2021). All these methods require the dataset to be extremely clearly described, which is difficult to generate in the context of Bangladesh.

Recently, satellite data have been utilized to predict the temperature in crop-growing areas (Prasad et al., 2021) (Danilevicz et al., 2021) (Jung et al., 2021). Because this method requires access to real-time satellite data, it would be inaccessible to most people. The precision of this method was additionally found to be insufficient. Some researchers also used the Neural Network (NN) approach to predict crop production, which might perform better than traditional ML methods (Minghua et al., 2012). However, NN is most common when working with multidimensional data. When the types of datasets are defined, the network model becomes more difficult to design, and more training time is required as the convergence time increases. It is also prone to slipping into the local minimal state.

Researchers have devised a way to predict crop yields at multiple spatial levels based on ML crop yield forecasts for regions. They developed a general ML workflow to show how proper regional agricultural yield forecasting can be in Europe. They predicted crop yields for 35 case studies, comprising nine nations that are major producers of six commodities (soft wheat, spring barley, sunflower, grain maize, sugar beets, and potatoes), to evaluate the validity and usefulness of regional predictions (Paudel et al., 2022). For the prediction of Irish potato and maize, authors collected data from multiple areas and analyzed it using RF, Polynomial Regression, and the SVR. The only variables employed as forecasters were rainfall and temperature. RMSE for RF was 510.8 and 129.9 for potato and maize, respectively, while *R* ^2^ was 0.875 and 0.817 for the same crop datasets, indicating that RF is the best model (Kuradusenge et al., 2023). Based on the previous 12 years’ data, researchers proposed an ML-based crop yield prediction in North China Plan. To find the best model, they investigate several ML algorithms on winter wheat and dry matter prediction (Wang et al., 2023).

In comparison to the existing works in the literature, we, in our study, (i) generate a learnable environmental dataset containing eight features to predict crop production; (ii) propose an ensemble ML algorithm using KNN, RF, and RR, called KRR; (iii) demonstrate that KRR produces better results than the other classical ML algorithms, such as SVR, NB, RR, RF, and CB; and (iv) design a recommender system that suggests suitable crops to grow in the next session. To this end, we deliver the parametric differences between our proposed paradigm and the studied interconnected crop production prediction works in **Table 2**.

**Table 2:** Comparison among the related works on crop production prediction.

Ref	Year	Dataset	Technique	Error/Score
(Cravero and Sepulveda, 2021)	2021	Big data	Classical ML and ensemble ML	Comparison charts
(Nandy and Singh, 2020)	2020	Collected data using multistage random sampling technique from 45 rural areas in West Bengal of India	RF and Logistic Regression (LR)	RF = 75.21% accuracy and 85.0% AUC and LR = 72.34% accuracy and 78.0% AUC
(Sharma et al., 2020)	2020	Collecting data from different sources	RF, DT, Bayesian network, SVM, NN, and GA	Comparison among the models
(Minghua et al., 2012)	2012	Historical agricultural product price data in China	NN	Prediction error 6.5% and 8.1% for different years
(Shehadeh et al., 2021)	2020	Bureau of Economic Analysis, U.S. Census Bureau	DT, LightGBM, and XGBoost	DT shows 93% accuracy, LightGBM 87%, and XGBoost 85%
(Charbuty and Abdulazeez, 2021)	2021	Private data generation	DT	Comparison among the models
(Sujjaviriyasup and Pitiruek, 2013)	2013	Thailand’s Pacific white shrimp export data	ARIMA and SVM	SVM (MAE 1504.52, RMSE 1978.79, and MAPE 11.22%)
(Lee and Moon, 2014)	2014	Yearly yield of apple	Kernel smoothing model	MAPE 5.7 and R2 is 1
Ours	2023	Self-generated dataset	KRR (proposed) and SVR, NB, RR, RF, and CB	KRR obtains highest results

## Proposed paradigm

Crop production prediction is a major concern for an agriculture-based country like Bangladesh because many prospective crops can be planted in a single season. Currently, the farmers choose the crops for plantation on their own knowledge, which might not be an effective prediction every time. Sometimes, it might give better production, and sometimes, not, which would then be very harmful to the economy of such an agriculture-based country. Moreover, the government necessitates predicting crop production to estimate crop amount for the upcoming year. We design an ensemble ML model to predict crop production based on the environment, cultivation area, and previous production parameters. We first gather real-world data records from different periods (1969–2021) of the diverse areas of Bangladesh and then propose an ensemble ML learning approach, called KRR, to accurately predict crop production on the basis of the environmental condition after inquiring about the most popular classical ML models. Using our KRR, the farmers can choose the best crops for the plantation, and the government can better estimate crop production for the next year. Notice that we do not find any such work to predict crop production in the Bangladesh context. Note that we discuss with a number of agriculturists to sort out the environmental factors related to the production of crops in Bangladesh. After that, we consider eight factors for predicting crop production, as illustrated in **Table 3**. The final dataset contains 7,000 samples of five categories of crops (Aus rice, Aman rice, Boro rice, potato, and wheat), each having 1,400 samples. If we want to add other crops in this system, then the same dataset should be generated and then we need to train the best-performing model as the procedure of ML training and testing.

**Table 3:** Short description of the attributes in the raw data records.

No.	Attribute	Short Description
1	Rainfall	Average rainfall of the months responsible for the specific crop
2.	Maximum Temperature	Average maximum temperature of the months responsible for the specific crop
3	Minimum Temperature	Average minimum temperature of the months responsible for the specific crop
4	Humidity	Average humidity of the months responsible for the specific crop
5	Wind Speed	Average wind speed of the months responsible for the specific crop
6	Cloud Coverage	Average cloud coverage of the months responsible for the specific crop
7	Bright Sunshine	Average bright sunshine of the months responsible for the specific crop
8	Aus Area	Total area of Aus cultivation including local area and High Yielding Variety (HYV) area in acres
9	Aman Area	Total area of Aman cultivation including local area and HYV area in acres
10	Boro Area	Total area of Boro cultivation including local area and HYV area in acres
11	Potato Area	Total area of potato cultivation including local area and HYV area in acres
12	Wheat Area	Total area of wheat cultivation including local area and HYV area in acres
13	Aus Production	Total production of Aus in tons
14	Aman Production	Total production of Aman in tons
15	Boro Production	Total production of Boro in tons
16	Potato Production	Total production of potato in tons
17	Wheat Production	Total production of wheat in tons

### Approach overview

**Figure 1:**
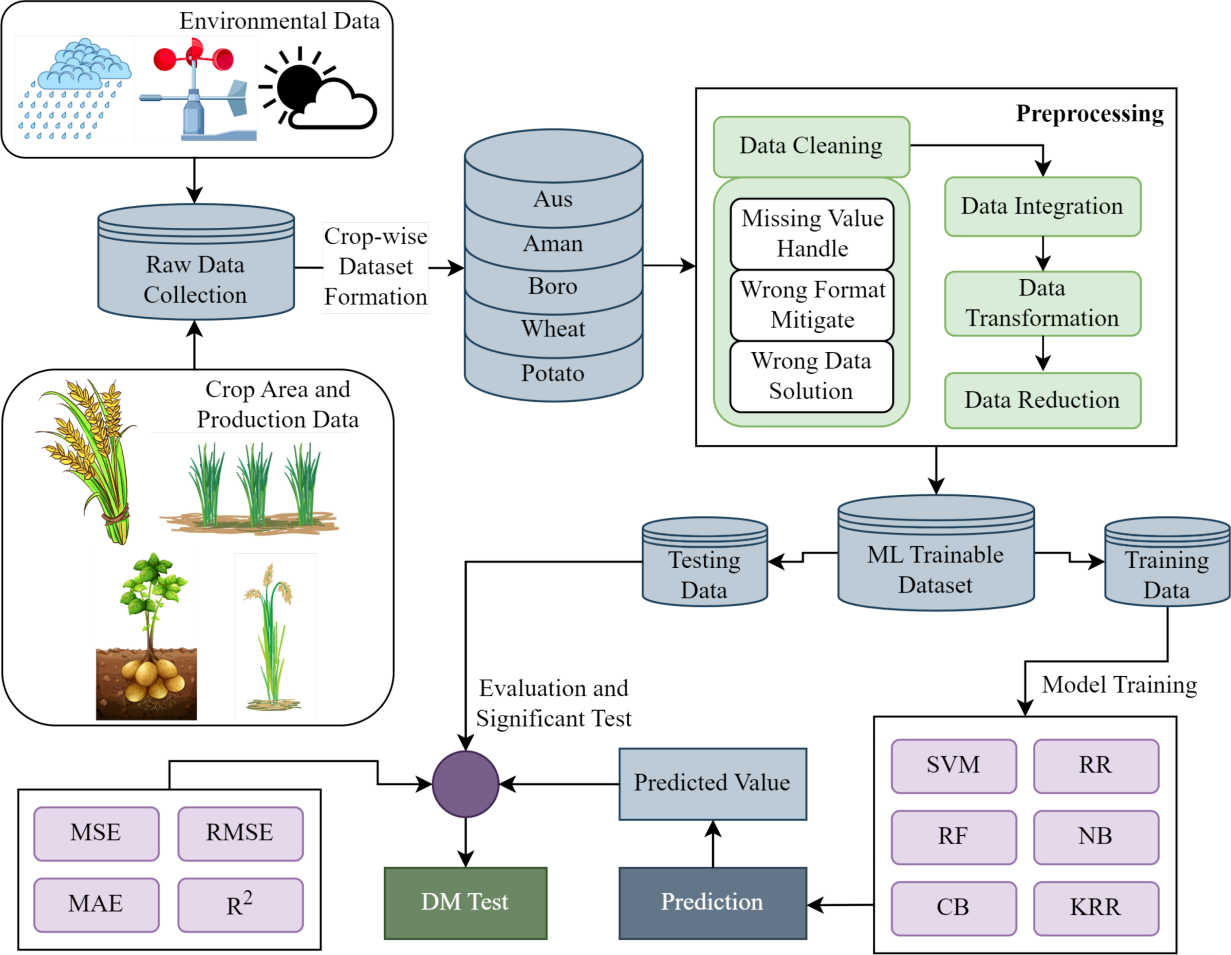
Proposed methodology for predicting crop production. Raw data collection, dataset preparation, data preprocessing, model development, crop production prediction, and model evaluation with the significant test are all carried out in synchronization throughout the entire methodology.

We illustrate the working steps of the proposed crop production and recommendation paradigm in **Figure 1**. The first stage is dataset preparation, which delivers a suitable data format for training and testing using the proposed ensemble learning and the existing investigated ML approaches after the necessary preprocessing and feature extraction. After that, the evaluation and analysis are performed based on the experimental results. Finally, the recommended system is presented for suggesting suitable crops for cultivating a specific region in the next season.

Top-down crop production prediction is depicted in the proposed methodology shown in **Figure 1**. After collecting raw data, we structure it crop-by-crop for the five regular crops in our research, jointly with the environmental variables during each of the crops’ relevant months. To create a dataset suitable for ML training, we handle missing values, mitigate wrong format and wrong data and modify raw data as required. In keeping with the standard ML practice, we split the final dataset into training and testing segments for the purposes of model training and testing with various evaluation methods. The ML models are trained independently using 80% of the training data and evaluated using the remaining 20%. After training and testing several models, we tabulate the evaluation outcome in terms of MSE, RMSE, MAE, and *R* ^2^. To determine the superiority of the proposed ensemble KRR, we conduct a DM significant test to compare it to the state-of-the-art benchmark ML models and determine its relative performance.

### Dataset preparation

We collect the raw data samples from four different agricultural organizations in Bangladesh, which are BMD, BADC, BRRI, and BBS from 1969 to 2021 of different seasons, as shown in **Table 4**. Kharif and Rabi are the two harvest seasons in Bangladesh for the majority of crops. The environment varies depending on the harvest season. The months responsible for crop production are considered when constructing the dataset for each crop. The specific crop’s weather information for the corresponding month is then provided. For example, Aus rice is harvested during the Kharif season, from June through August. It indicates that the monthly environmental data are regarded as weather data for Aus rice. Other crops’ samples are generated using the same manner, and the months corresponding to each season are listed in **Table 4**. In the final dataset, the data samples include 7,000 records of five categories of crops (Aus rice, Aman rice, Boro rice, potato, and wheat), each having 1,400 samples of different districts of Bangladesh. In particular, we prepare eight attributes (rainfall, maximum temperature, minimum temperature, humidity, wind speed, cloud coverage, bright sunshine, production area, and production amount) from a total of 17 original attributes to predict the production of a particular crop, as shown in **Table 3**. As the weather of the different crops is different for the month, we take the average of maximum temperature, minimum temperature, rainfall, humidity, wind speed, cloud coverage, and bright sunshine for each crop according to the month.

**Table 4:** Dataset description for different crops and environmental variables according to the season of the crops.

No.	Crop Name	Harvest Season	Data Duration	Weather Data
1	Aus rice	Kharif	1969 to 2021	June to August
2	Aman rice	Rabi	1969 to 2021	December to January
3	Boro rice	Kharif	1969 to 2021	March to May
4	Potato	Kharif	1969 to 2021	February to March
5	Wheat	Rabi	1969 to 2021	November to March

### Learning and evaluation

After generating the machine-learnable dataset, we split the dataset into training and testing sets. Then, we build the proposed ensemble ML (KRR) and investigated ML models (SVR, NB, CB, RF, and RR) using the training dataset and evaluate the trained models on the testing dataset to assemble the results. We consider four state-of-the-art performance indicators, i.e., mean absolute error (MAE), mean square error (MSE), root MSE (RMSE), and *R* ^2^ score to evaluate the proposed and investigated ML models. All the experiments (dataset preprocessing, model training, model testing, and result processing) are accomplished using the Python programming language.

## Methods and measurements

### Data preprocessing

We preprocess the collected raw data to make them machine-learnable datasets. As all of the values of our dataset are numeric, we need not label any data during data preprocessing. Besides this, we normalize the data lastly to make it more trainable.

The preprocessing steps are as follows.

#### Data cleaning

This step involves missing value handling, formatting, and wrong data handling. We handle missing values by replacing them with the mean of a feature, as illustrated in Algorithm 1. Because crop production of a country is a continuous process, environmental variable values follow a pattern. Missing values can be the mean of the previous and next values in our dataset. For particular features, we format all the data in a unique form, which helps improve the performance of the ML models (Stekhoven and Buhlmann, 2012).

#### Data integration

We consider three types of data, i.e., environmental parameters related to crop production, areas of cultivation, and crop production amount of a particular area. We collect these data from different government organizations. To prepare the learnable dataset, we integrate all data into a single dataset.

#### Data reduction

Unnecessary, duplicate, and junk data are harmful to the performance of the ML models (Royston et al., 2006; Benjelloun et al., 2007). To make the best ML learnable dataset, we remove unnecessary, duplicates, and junk values.

Algorithm 1 Missing value handling
**Input:** Raw data (***S***)
**Output:** Preprocessed dataset after missing value handling
1: **procedure** *MissingValueHandling*(***S***)
2:  **for** each attribute *S^a^* **do**
3: * m^a^* = mean (*S^a^*) [*m^a^* is the arithmetic mean of attribute *S^a^]*
4:  **for** each sample data *S_d_^a^* **do**
5:  **if** *S_d_^a^* is missing **then**
6:  *S_d_^a^*:=*m^a^*
7: ** end if**
8: ** end for**
9:  **end for**
10: **end procedure**

#### Data normalization

Finally, we normalize the entire dataset to integer values to fit into the ML algorithms. We employ the classical min–max normalization technique to normalize the dataset.

### Training models

In this section, we recapitulate the working principles of the investigated ML and ensemble ML approaches (SVR, NB, RR, RF, and CB) and present our proposed ensemble ML paradigm (KRR). We select five benchmark ML models instead of all ML algorithms in a strategy. ML algorithms can be classified based on architecture and working procedure. We choose SVM as the representative of the distance-based ML algorithm, RR from the group of regularization ML algorithms, RF as the representative of the bagging ensemble algorithm, NB as the member of the Bayes theorem means probability-based ML algorithm, and CB as the representative of boosting ensemble algorithm. We select those five algorithms to represent all ML algorithms in our analysis, train them individually using our dataset, and measure their performances to compare with the proposed ensemble KRR.

#### SVR

SVR is a supervised ML algorithm that is a useful technique for both data classification and regression (Somvanshi et al., 2016). In regression, the data are separated into training and testing sets. Each instance in the training set contains one target value (class label) and several attributes named as the features (observed variables). The goal of SVR is to produce a model (based on the training data) that predicts the target values of the test data given only the test data attributes (Shang et al., 2016). According to the characteristics of our dataset, we use the linear SVR approach for predicting different agricultural crop production rates.

#### NB

NB is one of the most efficient and effective inductive ML algorithms (Ratanamahatana and Gunopulos, 2003) (Panda and Patra, 2008). This uses the Bayes theorem to calculate the probability and then form a prediction. The basic insight of Bayes’ theorem is that when new data are introduced, the probability of an event may be changed. The NB model is simple to implement and it does not require sophisticated iterative parameter estimation, making it perfect for large datasets (Razzaghi et al., 2016).

#### RR

RR is a model optimization technique (Tavares et al., 2021). It estimates the coefficients of multiple regression models under conditions of high correlation between linearly independent variables. This model is also known as a regularization model and uses the *L*2 regularization process. It has been applied in various disciplines, including agricultural data, engineering, chemistry, and econometrics. RR creates a new matrix by adding a ridge parameter (*k*) from the identity matrix to the cross-product matrix. The reason it is known as *ridge regression* is that the correlation matrix’s diagonal of one can be compared to a ridge. Overfitting is a problem that RR solves since squared error regression by itself can distinguish between significant and insignificant features, using all of them instead, resulting in overfitting (Garriga et al., 2017). RR introduces a small amount of bias in order to match the model to the actual values of the data. However, it does not have the ability to do feature selection and the final model includes all predictors. It swaps variance for bias and decreases coefficients toward zero.

#### RF

RF is an ensemble ML classifier that uses randomness to create a group of independent and non-identical DTs (Provost et al., 2016). This algorithm is used for both classification and regression purposes and it is a combination of tree predictors. Each DT has a random vector as a parameter, determines the feature of samples at random, and selects the training dataset from either a subset of the dataset at random (Bradter et al., 2013). Whenever a random selection of features is used to split each node, the error rates are equivalent to Ad boost, but they are more robust in terms of turbulence (Shakoor et al., 2017). RF is a highly flexible and easy-to-use ML algorithm that produces, even without hyper-parameter tuning, a great result most of the time. In this work, we use RF for the regression aspect of this algorithm based on our necessity. We successfully achieve a very high accuracy upon implementation of our dataset using this RF regression. Python’s scikit-learn has a helpful tool for this that quantifies the relevance of a feature by looking at how much inaccuracy is minimized across all trees in the forest by tree nodes using that feature (Grange and Hand, 1987). Deep DTs might suffer from overfitting but RF prevents overfitting most of the time. It creates random subsets of the features and builds smaller trees using these subsets, and afterward, it combines the sub-trees.

#### CB

CB is an ML algorithm for gradient boosting on DTs. Gradient boosted DTs are a powerful tool for classification and regression. This algorithm is developed by Yandex researchers and engineers and it is the successor of the MatrixNet algorithm. It is widely used for ranking tasks, forecasting, and making recommendations (Hancock and Khoshgoftaar, 2020). This supervised algorithm is used both for classification and regression purposes. CB is a special type of boosting algorithm with much less prediction time for its symmetric tree structure. However, it is sensitive to its hyperparameter tuning.

#### The proposed ensemble ML approach

**Figure 2:**
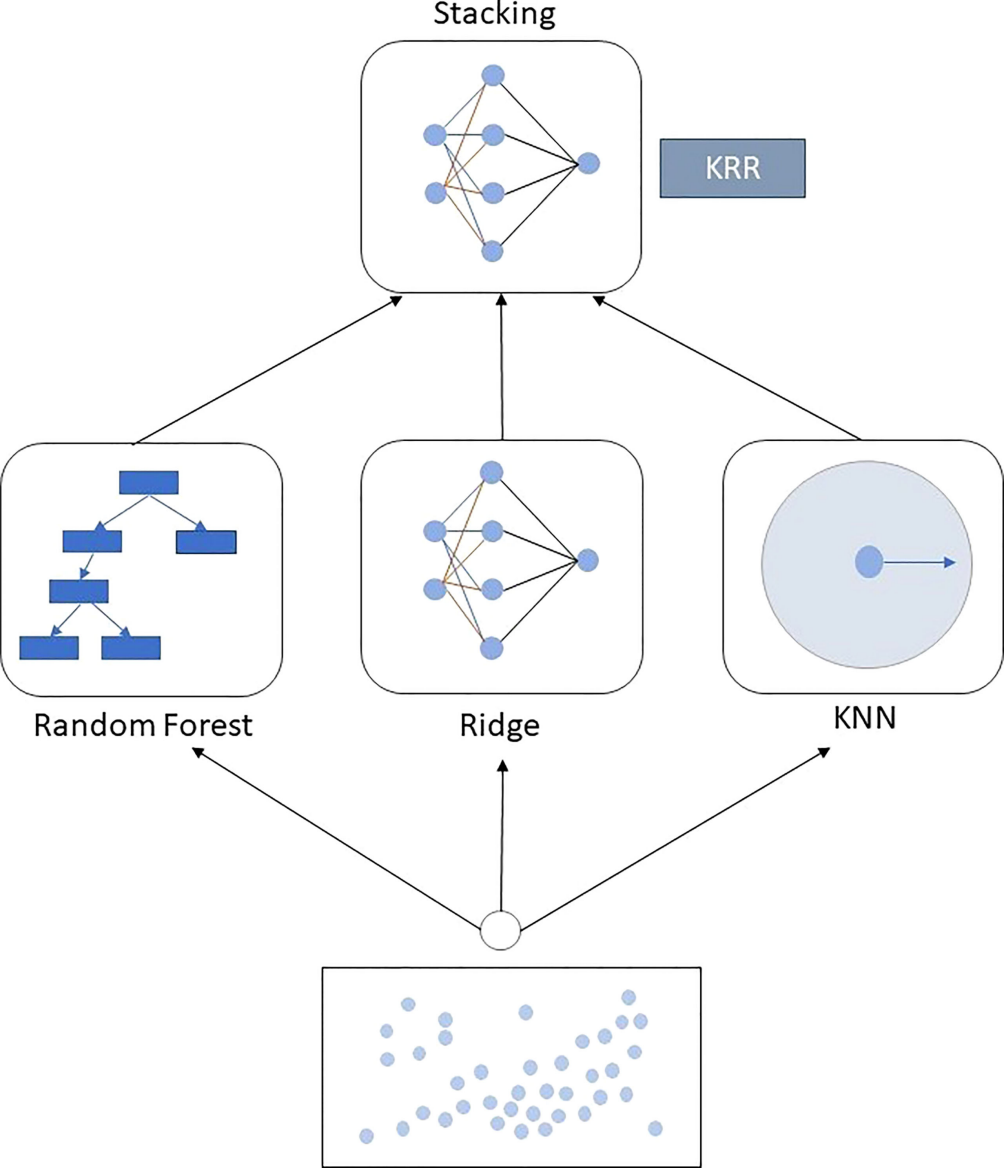
Block diagram of the proposed KRR approach. KRR is built with the three mostly used ML algorithms: KNN, RF, and RR, using the blending ensemble strategy.

The main purpose of introducing an ensemble regressor is to reduce the variance of the data during model training (Sagi and Rokach, 2018). It helps to fit the data to the models, and the model can predict more accurately. In the proposed KRR ensemble method, we use a distance-based algorithm KNN, a regularization method RR, and a tree-based ensemble RF. The KNN model is simple to implement and works well with non-linear data. Because it does not require calculating any fixed parameters or values, fitting the model also takes little time. The KNN algorithm makes predictions about the significance of new data points based on their “feature similarity”. A score is given to the new point based on how similar it is to the points used for training. RR is good for preventing overfitting, which adds one additional element to the cost function of linear regression. The primary reason these penalty terms are included is to ensure regularization, or the reduction of model weights to zero or close to zero so that the model does not overfit the data. Nonlinear parameters do not affect the performance of an RF, unlike curve-based techniques. As a result, if the non-linearity between the independent variables is high, RF may beat other curve-based methods. It is usually robust to outliers and can handle them automatically. It does not require feature scaling (standardization and normalization) because it employs a rule-based method rather than distance calculation. That is the reason for creating the new ensemble model using the algorithms that can handle overfitting by themselves, with no need for extra preprocessing when needed during training and testing. A second-order ensemble strategy called blending is used to construct this KRR regression method. Blending ensemble ML methods find the best combination of the predictors from the three ML algorithms (KNN, RR, and RF) and form an ensemble regressor for better prediction (Farooq et al., 2021). The blending process is the same as the stacking ensemble procedure, but it has some unique differences. Stacking uses out-of-fold prediction for the training set of the next layer in the meta-model. On the other hand, our blending uses a validation set (10%–15% of the training data) to train the next layer in the meta-model. KRR combines the mapping functions learned by the contributing members. Our proposed KRR is the combination of the hyperplanes of KNN, RF, and RR. The working procedure and function mapping of KRR are shown in **Figure 2**.

The working principle of KRR is different from the KNN, RF, and RR, which are the building blocks of the ensemble approach. The proposed KRR and RF are both ensemble methods, where KRR is a blending ensemble where the building blocks work individually to find the main output. RF is a bagging ensemble where data are mainly divided into several bags to train the individual tress to formulate the result. The benchmark models sometimes fall into overfitting problems, and due to the high variance of the data points, the performance of the models falls in some cases, but the proposed method outperforms in this case by its tolerance and flexibility of learning from the dataset.

Our deployed KRR can be a solution to this type of regression issue for better performance and the best fitting of datasets. The criteria for adopting the proposed scheme KRR to another dataset are very easy. As with the traditional ML training and testing process, the training data must fit the KRR architecture and then evaluated by the remaining testing data. The KRR architecture is already described above. However, hyperparameter tuning of the building block ML algorithms of KRR can bring a better result when adopted with other datasets.

##### Complexity of proposed ensemble KRR

The complexity of KRR can be written into two steps. In the first step, the complexity of stacking architecture forms and then the individual algorithm’s complexity is added one by one in the next step as follows: The complexity of the first step is O(B(C + R)), where R represents the number of replacements, the number of bags of the dataset is B, and C represents the number of classifiers in the ensemble algorithm. In the second step:

1. Complexity KNN is O(nd), where n is the number of training examples, and d is the number of features.
2. Complexity of RR is O(*n* ^3^), where n is the number of data.
3. Random Forest of size T and maximum depth D (excluding the root) is O(T.D).

### Model evaluation

The classical and ensemble ML algorithms and our proposed ensemble ML scheme are applied to predict crop production. The training data train these approaches, and the model learns the data sequences and then forms a prediction. The performance of the ML models is calculated using four evaluation metrics, i.e., MAE, MSE, RMSE, and *R* ^2^. MSE can be defined as the absolute value of the difference between the predicted and actual value. Using MSE in regression will penalize large errors more than small ones if we assume that the target follows a normal distribution. The MSE is calculated as:


$$MSE=\frac{1}{n}\sum_{i=1}^{n}{\left(Y_{i}-{\hat{Y}}_{i}\right)}^{2}$$

MAE indicates how big of an error we may expect on average from the prediction (Morales and Villalobos, 2023). MSE indicates how close it is to a set of points. It accomplishes this by squaring the distances between the points and the regression line (these distances are the errors). Squaring is required to eliminate any negative signs. The MAE is calculated as:


$$MSE=\frac{1}{n}\sum_{i=1}^{n}\left|Y_{i}-{\hat{Y}}_{i}\right|$$

RMSE is the standard deviation of the residuals (prediction errors) (Glennie and Lichti, 2010). Residuals are a measure of how far the data points are from the regression line; RMSE is a measure of how to spread out these residuals. In other words, it reveals how strongly the data are aggregated around the line of best fit. The RMSE is calculated as:


$$MSE=\sqrt{\frac{1}{n}\sum_{i=1}^{n}{\left(Y_{i}-{\hat{Y}}_{i}\right)}^{2}}$$

*R* ^2^ is a statistical measure of how much variation in a dependent variable can be explained by variation in the independent variables. The main objective of this score is to predict future results based on existing data. The extent to which the model can reproduce observed results is quantified by this measure, which is based on the fraction of the total variation in outcomes that can be attributed to the model. *R* ^2^ is calculated as:


$$R^{2}=1-\frac{{\sum (Y_{i}-\hat{Y}}_{i}{)}^{2}}{{\sum (Y_{i}-Y}_{i}{)}^{2}}$$

In the above equations, the *Y_i_*indicates the actual value, *Y* ^ˆ^ *_i_*indicates the predicted value, *Y*¯ indicates the means of the *Y* values, and *n* is the total number of samples.

## Experiment and result analysis

### Experimental setting

We use Python’s *scikit-learn* tool to construct the proposed ensemble ML scheme (KRR) as well as the investigated classical and ensemble ML models (SVR, NB, RR, RF, and CB). We consider the actual vs. predicted curve and error metrics (MAE, MSE, RMSE, and *R* ^2^) as the evaluation parameters of the trained ML models. We use supervised methods to predict crop production, where the dataset contains eight features and the target is the amount of crop production in a certain area. We take the average results of experiments in three phases, such as 80:20, 50:50, and 30:70 training and testing ratio, where each phase has 10 trials.

To get better performance, we tune the hyperparameters of the proposed ensemble ML scheme as well as the investigated classical and ensemble ML models. The same hyperparameters give a better result for almost all experiments. In particular, SVR gives better results with the linear kernel when *c* = 100 and gamma is *auto* while we use 10-fold cross-validation to find the value of *gamma* and *c*. Gaussian NB achieves a better result in all cases with the hyperparameters i.e., *estimator* = *model*, *param_grid* = *params_nb*, and *cv* = *cv_methods*. RF finds *n_estimator* = 20, and *random_state* = 42 in all cases for better performance. For all experiments, RR gives maximum performance when *alpha* = 0.01. CB model gives a better result when *estimator* = *model_cvr*, *cv* = 2 n_jobs = −1, and *learningrate* = 0.05. Our proposed model KRR achieves high accuracy with a low error rate with the hyperparameters *alpha* = 0.01, *n_estimator* = 10, and *random_state* = 42 for almost all experiments.

### Result analysis on Aus rice production

**Figure 3:**
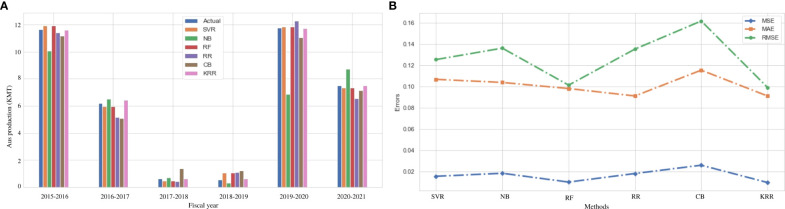
Comparison of the actual and predicted values of Aus rice production and error rating of the investigated and proposed ML models (SVR, NB, RF, RR, CB, and KRR) in each fiscal year from 2015 to 2021. **(A)** Actual vs Predicted bar chart for Aus. **(B)** Line chart for the error rating of Aus production prediction.

Aus is considered one of the major crops in Bangladesh. This type of rice is closely related to indica-type rice but it has a distinct genetic group (Chakravarthi and Naravaneni, 2006). Still, this variety is cultivated under environmental stress conditions in Bangladesh and India (Berger et al., 2004). The value of Aus production varies according to the environment and the region of cultivation. **Figure 3A** represents the actual vs. predicted rice production in every fiscal year from 2015 to 2021 in the Dinajpur zone of Bangladesh. The *x*-axis symbolizes the fiscal year, while the *y*-axis reflects rice production (both actual and predicted). It clearly indicates that our proposed algorithm outperforms the other traditional ML and ensemble ML algorithms. We also evaluate MAE, MSE, RMSE, and *R* ^2^ to measure the model’s goodness of fit in predicting rice production in **Figure 3B**, which demonstrates that the KRR model fits better than the other models we investigate. **Table 5** illustrates that KKR has less mistakes, such as 9.11% MAE, approximately 1% MSE, and 9.17% RMSE, than the others.

**Table 5:** Error ratings using the investigated and proposed ML approaches for predicting Aus rice production using different metrics and *R* ^2^ score.

Model	MAE	MSE	RMSE	*R* ^2^
SVR	0.107	0.016	0.125	0.980
NB	0.104	0.019	0.136	0.980
RF	0.098	0.010	0.101	0.990
RR	0.091	0.018	0.135	0.980
CB	0.115	0.026	0.161	0.970
KRR	0.091	0.009	0.099	0.990

From **Figure 3A**, it can also be observed that there is no linear relationship between the area and environmental data, and Aus rice production. The weather conditions in Bangladesh vary from year to year, and natural disasters may occur. In August 2017, during the Kharif harvest season, an uncertain flood occurred in Dinajpur zone (Das and Rahman, 2018). It damaged the crops and interrupted the production cycle. Our proposed KRR performs effectively during this period, which demonstrates its adaptability to the uncertainty in the environmental data. Owing to a lack of soil fertility and improper management of soil carbon, the post-flood effects on Aus production continue throughout the subsequent growing seasons (Siddique et al., 2022). In this uncertain situation, the proposed KRR performs better than other models. This type of prediction is critical for farmers as well as individuals who depend on harvesting for a living. Such future production prediction aids in the care of alternative solutions to ensure food and industrial raw materials.

### Result analysis on Aman rice production

**Figure 4:**
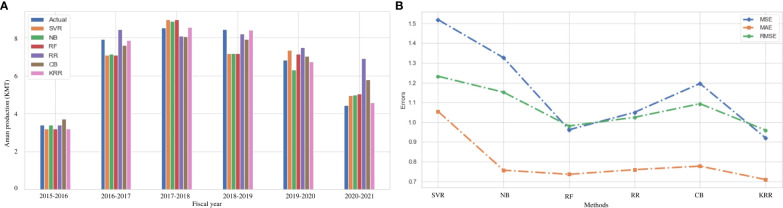
Comparison of the actual and predicted values of Aman rice production and error rating of the investigated and proposed ML models (SVR, NB, RF, RR, CB, and KRR) in each fiscal year from 2015 to 2021. **(A)** Actual vs Predicted bar chart for Aman. **(B)** Line chart for the error rating of Aman production prediction.

Aman rice is grown in Bangladesh during the winter (rabi) season. The cultivation of Aman rice is strongly linked to the environment. **Figure 4A** illustrates the actual and predicted values using the investigated and proposed ML algorithms. The performance of our proposed algorithm KRR reaches maximum accuracy for each fiscal year. In terms of error measurement parameters, both our proposed KRR and the RF models have the same *R* ^2^ score. However, our KRR obtains better MAE, MSE, and RMSE than RF and other models, as shown in **Table 6**. **Figure 4B** indicates that our KRR is the best-fit model compared to others. Changes in maximum temperatures have had a significant impact on crop yield in Bangladesh. However, temperature changes confirm that maximum temperature raises the risk for Aman rice while minimum temperature reduces yield variability. Rainfall has increased the risk of Aman rice (Sarker et al., 2019). The great news is that environmental factors in Bangladesh are now changing within a range that allows Aman rice to adapt to the environment. In recent years, Aman rice has been consistently produced because of its versatility (Chakrobarty et al., 2021). **Figure 4A** shows the consistency of Aman rice production, and in most of the cases, the proposed KRR performs better. The authority can benefit from this method in their long and short plan for food supply in the future.

**Table 6:** Error ratings using the investigated and proposed ML approaches for predicting Aman rice production using different metrics and *R* ^2^ score.

Model	MAE	MSE	RMSE	*R* ^2^
SVR	1.055	1.510	1.233	0.790
NB	0.757	1.326	1.152	0.860
RF	0.736	0.962	0.981	0.900
RR	0.759	1.050	1.025	0.890
CB	0.778	1.196	1.093	0.870
KRR	0.709	0.921	0.959	0.900

### Result analysis on Boro rice production

**Figure 5:**
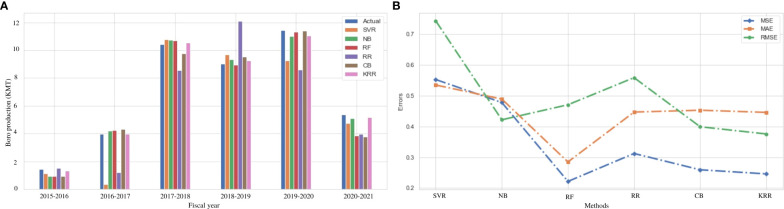
Comparison of the actual and predicted values of Boro rice production and error rating of the investigated and proposed ML models (SVR, NB, RF, RR, CB, and KRR) in each fiscal year from 2015 to 2021. **(A)** Actual vs Predicted bar chart for Boro. **(B)** Line chart for the error rating of Boro production prediction.

Boro rice is cultivated in the Kharif season, which has a vital impact on the total rice production in Bangladesh. **Figure 5A** illustrates the actual vs. predicted bar chart for the Boro production from 2015 to 2021, while **Figure 5B** indicates that KRR is the best-fit model compared to others. **Table 7** indicates that the performance using RF is better than KRR in respect of MAE and MSE. However, RMSE and *R* ^2^ are good in KRR. In summary, the average performance of KRR is better than the other models.

**Table 7:** Error rating representation using the investigated and proposed ML approaches for predicting Boro rice production using different metrics and *R* ^2^ score.

Model	MAE	MSE	RMSE	*R* ^2^
SVR	0.535	0.553	0.744	0.960
NB	0.489	0.478	0.422	0.960
RF	0.285	0.222	0.471	0.980
RR	0.447	0.312	0.559	0.970
CB	0.453	0.259	0.399	0.980
KRR	0.446	0.246	0.376	0.990

To this end, we investigate three major rice variations in this part to predict their production concerning the environmental conditions. Given the analyses, we can deduce that our proposed model KRR performs better in predicting different rice production in Bangladesh. Boro rice needs extra irrigation for cultivation. The average production of Boro rice is expected to decrease by over 20% in 2050 and by 50% in 2070 as a result of climate change (Basak et al., 2010). It has been determined that an increase in both the maximum and minimum temperatures is the primary cause of a reduction in yield. Rainfall pattern changes during the growing season have also been observed to impact rice production and irrigation needs. Using the proposed KRR, researchers can track environmental factor changes and then take the necessary steps to select an alternative rice variety or predict the production of the new variety.

### Result analysis on potato production

**Figure 6:**
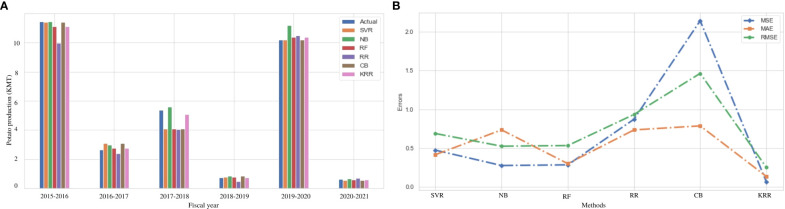
Comparison of the actual and predicted values of potato production and error rating of the investigated and proposed ML models (SVR, NB, RF, RR, CB, and KRR) in each fiscal year from 2015 to 2021. **(A)** Actual vs Predicted bar chart for Potato. **(B)** Line chart for the error rating of Potato production prediction.

In Bangladesh, potato farming takes place throughout the winter season. Sandy loam soils can produce more potatoes than other types of soil (Faraji et al., 2017). In terms of productivity and internal demand, potatoes are a popular crop in Bangladesh. As a result, predicting potato production has a significant influence on the economy. **Figure 6A** demonstrates a comparison among the actual and predicted values using the investigated and proposed ML approaches, which shows that our proposed KRR approach offers superior prediction in almost all cases. According to **Table 8**, our proposed approach KRR outperforms the other investigated ML algorithms in all error measures. In particular, KRR predicts potato production with a minimum MSE of 6.3%. **Figure 6B** illustrates that KRR is the best-fit model to predict potato production. Potato production in Bangladesh is still at a satisfactory level but it swings to change of environment (Hossain and Abdulla, 2016). In some consecutive years, the production goes down due to heavy cold and attack of unexpected diseases on potato (Islam et al., 2022). To predict production in this kind of uncertain situation, the proposed KRR can be a good solution for the agriculture domain people.

**Table 8:** Error ratings using the investigated and proposed ML approaches for predicting potato production using different metrics and *R* ^2^ score.

Model	MAE	MSE	RMSE	*R* ^2^
SVR	0.416	0.474	0.688	0.960
NB	0.735	0.275	0.525	0.970
RF	0.299	0.284	0.533	0.980
RR	0.735	0.873	0.934	0.930
CB	0.787	2.144	1.464	0.840
KRR	0.134	0.062	0.250	0.990

### Result analysis on wheat production

In Bangladesh, the production of wheat is decreasing on average by 0.44% each year. People are cultivating different crops instead of wheat for more benefits and a higher production rate. The prediction of the production of wheat can improve the production rate of wheat. We use the same ML and ensemble ML algorithms to predict wheat production in the Dinajpur zone of Bangladesh.

**Figure 7:**
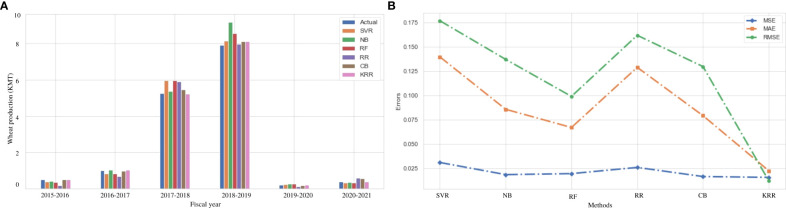
Comparison of the actual and predicted values of wheat production and error rating of the investigated and proposed ML models (SVR, NB, RF, RR, CB, and KRR) in each fiscal year from 2015 to 2021. **(A)** Actual vs Predicted bar chart for Wheat. **(B)** Line chart for the error rating of Wheat production prediction.

**Figure 7A** indicates a comparison among the actual and predicted values of wheat rice production using the investigated (SVR, NB, RF, RR, and CB) and proposed (KRR) ML models in each fiscal year from 2015 to 2021. It demonstrates that the performance of our proposed KRR is better than other ML models. In terms of other error metrics, KRR achieves the best result than the other investigated ML approaches, as illustrated in **Table 9** and **Figure 7B**. Wheat production in South Asia climbed from 15 million tons in the 1960s to 95.5 million tons in 2004–2005. It still needs to increase at a rate of 2%–2.5% every year till the middle of the 21st century (Chatrath et al., 2007). Because there is little scope for growing wheat field areas, the main task will be to crack the yield barrier utilizing practical genetic and morphological techniques. Other issues are unique to the highly productive rice–wheat farming system prevalent in the Indo-Gangetic plains. Though the production is at a low level, the 2017–2078 and 2018–2019 time periods have broken records. Previously, we discussed that the damage of the Aus rice due to flood plays a vital role in this segment (Das and Rahman, 2018). People engaged more in cultivating wheat to recover the damage to the economy in the period. It indicates that cultivating more wheat can be a solution to increase the amount of wheat production, which leads the agricultural economy in another direction.

**Table 9:** Error ratings using the investigated and proposed ML approaches for predicting wheat production using different metrics and *R* ^2^ score.

Model	MAE	MSE	RMSE	*R* ^2^
SVR	0.139	0.031	0.177	0.960
NB	0.086	0.019	0.137	0.970
RF	0.067	0.019	0.099	0.980
RR	0.129	0.026	0.162	0.960
CB	0.079	0.017	0.129	0.980
KRR	0.023	0.016	0.013	0.990

To summarize, we can state that, on average, our proposed model KRR outperforms the others in predicting crop production for all crops considered in this work. Regarding MSE, MAE, RMSE, and *R* ^2^, the proposed KRR performs better. From **Tables 5** to **9**, we can clearly differentiate the performance of each model for prediction. In almost all cases, the MSE, MAE, and RMSE values of KRR are smaller than those of the other models, which indicates that KRR shows minimum error in the case of prediction compared to other ML models. However, the *R* ^2^ value of the KRR is larger than the other models in the above-mentioned tables. It also creates a comparison among the models that KRR is a better fit to the dataset than existing benchmark ML models. To find the superiority of KNN, the DM significant test is also performed below.

### Significant test on the superiority of the proposed ensemble ML model

#### DM test

The DM test is one of the most used significant test procedures to compare the robustness of the best method in prediction. This is an asymptotic z-test of the hypothesis that calculates the loss difference (Diebold, 2015). In this study, we consider the null hypothesis as *H0*, i.e., the loss difference of model A is lower than or equal to that of model B. Note that the hypothesis rejection means model B is significantly more accurate than model A. In every hypothesis, testing model B is our proposed KRR ensemble model.

#### Significant test result

We use the DM test to find the significance of our proposed ensemble algorithm KRR compared to the other investigated ML models. We evaluate this test for the Aus rice, Aman rice, Boro rice, wheat, and potato data.

**Table 10** illustrates the DM values of different models compared to our proposed KRR. For almost all of the cases, our proposed KRR shows 5% significance over other ML models.

**Table 10:** DM value with significance for each investigated ML model compared to our proposed KRR ensemble model in terms of DM and *p*-values.

Investigated Model	Aus Rice (DM Value)	Aman Rice (DM Value)	Boro Rice (DM Value)	Potato (DM Value)	Wheat (DM Value)
SVR	23.041*	16.323*	17.554*	12.253*	13.862*
NB	22.060*	15.542*	5.884**	13.870*	11.960**
RF	2.561***	2.870***	22.021*	10.530**	9.532**
RR	4.292***	4.454***	0.460***	11.984**	12.933**
CB	6.160**	5.920**	14.744*	10.953**	8.192**

Observation represents the algorithm’s DM value of Aus rice, Aman rice, Boro rice, wheat, and potato while *, **, *** represent the significance level according to the p-values of the test. * represents 1% significance, ** represents 5% significance, and *** represents 10% significance of our proposed model against the investigated algorithms.

## Crop recommender system

**Figure 8:**
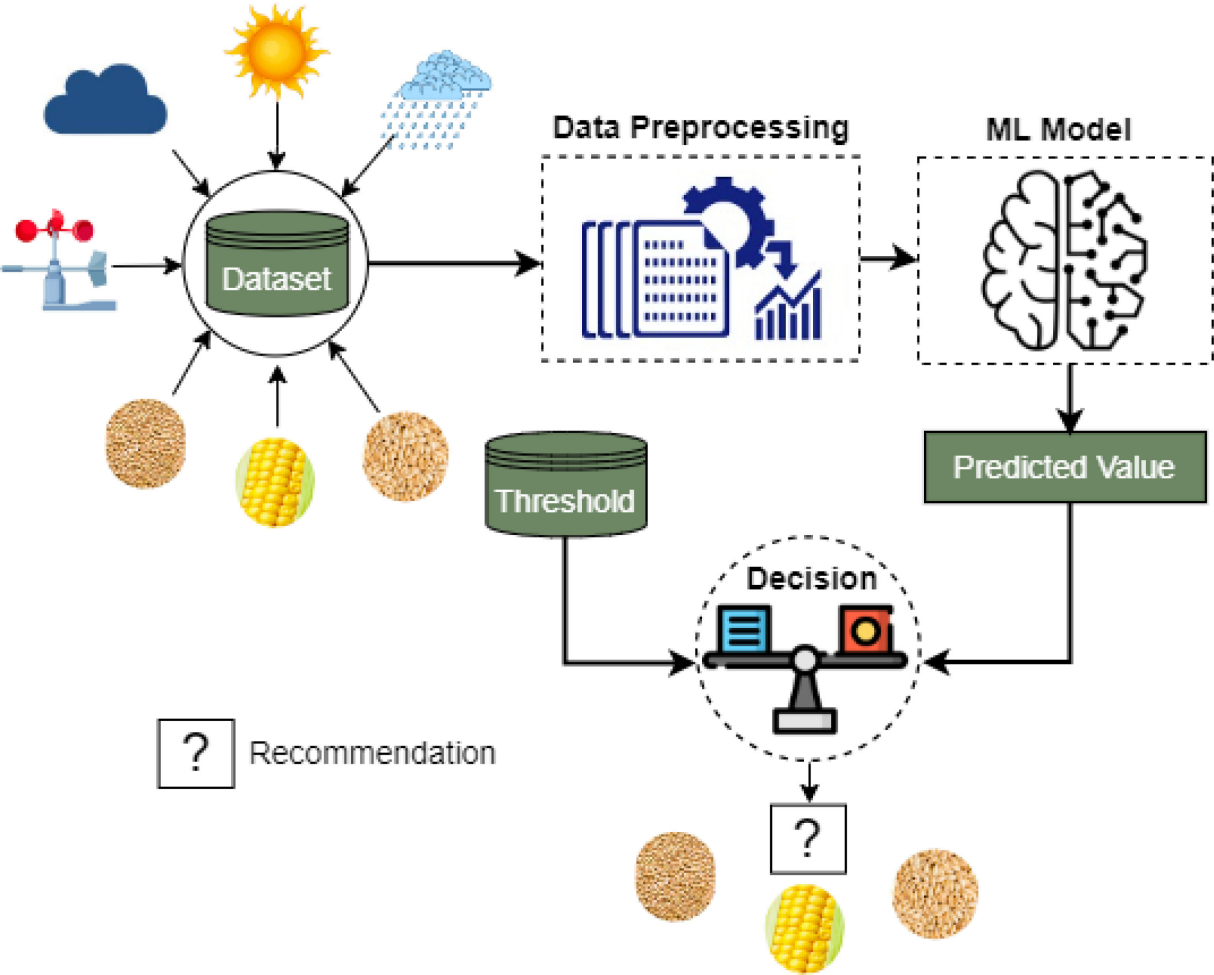
Block diagram of the proposed crop recommender system. This recommender system employs our proposed pre-trained KRR ML model to predict the production values of the test samples of different crops. After that, a suitable crop to grow is recommended using the predicted production values and the expert-defined threshold.

Besides crop production forecasting, crop recommendation is a vital part of such a study. Suitable crops in suitable land can boost the production of any crop (Bhullar et al., 2023). Finding the best crops for the appropriate land is a challenging task. A complex analysis of the environmental variables and production rate is required to recommend a land for production (Van Ittersum et al., 2013). Every area has a unique value of environmental variables. Considering the standard variables as threshold values (collected from expert agriculturists), we propose to recommend crops for any specific area. We provide the block diagram in **Figure 8**. First, the model is trained on the environmental and area data, and then, based on the environment and area data of the current year, the estimated production is predicted for each crop. Next, the production of the individual crops is compared with the threshold value of the crop for any specific area. If the production satisfies the criteria, then the crop will go to the list of recommendations. The threshold value is to be estimated by the associated local authority, which can be changed according to changes in the status of the area, demand, policy, and also environmental conditions. Comparing the crops of the same harvesting period, the model will recommend the right crops for the right place. We also introduce the pseudocode to recommend the crops for the land in Algorithm 2. We now discuss every step in the recommender system as follows.

### Dataset creation

Dataset is one of the significant components of the recommendation system (Zhang et al., 2018). A dataset should train the model used for prediction and recommendation to understand the nature of the system. Then, it can predict and recommend the outcome. In this recommendation system, environmental, area, and production data of the crops are taken into consideration. Five main crops of Bangladesh are considered here as the source of data. The data collection procedure and preprocessing are discussed in detail in the *Methods and measurements* section.

### Model development and prediction

This stage is one of the crucial parts of the crop recommendation system. The preprocessed dataset is used to train the ML models, and the model predicts the production of different crops. The model makes specific crop predictions based on the area, previous production, and environmental data related to the specific crop production period. Our proposed high-performance model KRR described in the *Proposed paradigm* section is considered as the potential ML model in the recommendation system.

Algorithm 2 Crop recommendation
** Input:** *θ* **(Train model);** *ξ* **(Environmental data);** *α* **(Land area);** *ν* **(Crops’ names);** *τ*
**(Threshold value)**
**Output: Recommended crop**
1: **procedure** *CropRecommendation (θ, ξ, α, ν, τ)*
2:  **for** each *ν* **do**
3:   *P^ν^
* ← *Predict*(*θ,ξ,α*); [*P^ν^
* Predicted production amount of *ν]*
4:  **end for**
5:  **if** *P^ν^
* ≥ *τ* **then**
6:   return *ν*
7:  **end if**
8: **end procedure**

### Threshold value selection

We consider the standard environmental data as a threshold for the recommendation. In a specific area, crop production depends on environmental properties like temperature, rainfall, wind speed, and sunshine. The environmental variables can be predicted by our proposed model besides the crop production prediction. Threshold values are taken from the local agriculture office and set to our model for the recommendation.

### Crop recommendation

It is the final step of the recommender system. The model predicts the production for an area, and we set the threshold for this system. The threshold is compared with the predicted environmental data, and the crops’ production is considered the main element of recommendation. The recommendation is reached by comparing the threshold with the predicted data for each season’s crop. The top match is the recommended crop for the season in the area. The input of this system is the season and the list of crops, and the output is the recommended crop among the selected crops. As an extension of this work, in the future, a mobile application will be developed that will help farmers gain easy access to this system. However, in this current state, they can use it with the help of experts with a technical knowledge of putting the inputs and synchronizing with this system.

## Conclusion

In this work, we have focused on designing a learnable dataset on agricultural crop production prediction from different agricultural research organizations as well as the meteorological department of Bangladesh. The analysis is first performed using five popular classical ML algorithms as well as ensemble ML algorithms. Then, we proposed an ensemble algorithm, called KRR, to better predict crop production. After evaluating all the algorithms, we have found that our proposed ensemble method KRR outperforms the investigated traditional ML and ensemble ML algorithms. In particular, KRR shows minimum errors and a maximum *R* ^2^ score compared to that of the investigated ML approaches. We have also provided a DM test to demonstrate the superiority of our proposed KRR approach over the existing ML approaches. The final result also indicates that the production of rice is increasing day by day, and the production of potatoes is also increasing at a significant rate while the production of wheat is decreasing every year. We have also provided a crop recommender system that recommends the most suitable crops to be cultivated on a particular land in the upcoming season.

## Limitations

This work focused on predicting major crops than the minor crops due to the lack of available data in the study area. Some factors such as soil properties, production cost, and market price, the data collection process of which is difficult and time-consuming, were not considered.

## Future work

In the future, we will gather more data related to this study and analyze deep learning methodology that can help to select the appropriate crops for the right land more correctly. Local market and wholesale market price analysis will also be performed to select the crops for a specific region. For a more complete picture, researchers plan to include both modern and traditional crops in their future analyses and selections. Additionally, a system based on mobile applications can be created to guarantee that farmers have easy access to the system’s information.

## Data availability statement

The original contributions presented in the study are included in the article/supplementary material. Further inquiries can be directed to the corresponding authors.

## Author contributions

MH and MM: Conceptualization. MU and MA: Methodology. SK: Software. SM and YN: Validation and formal analysis. All authors contributed to the article and approved the submitted version.
